# The more the merrier? Increasing group size may be detrimental to decision-making performance in nominal groups

**DOI:** 10.1371/journal.pone.0192213

**Published:** 2018-02-27

**Authors:** Ofra Amir, Dor Amir, Yuval Shahar, Yuval Hart, Kobi Gal

**Affiliations:** 1 Technion - Israel Institute of Technology, Haifa, Israel; 2 Ben-Gurion University, Beer-Sheva, Israel; 3 Harvard University, Cambridge, MA, United States of America; Southwest University, CHINA

## Abstract

Demonstrability—the extent to which group members can recognize a correct solution to a problem—has a significant effect on group performance. However, the interplay between group size, demonstrability and performance is not well understood. This paper addresses these gaps by studying the joint effect of two factors—the difficulty of solving a problem and the difficulty of verifying the correctness of a solution—on the ability of groups of varying sizes to converge to correct solutions. Our empirical investigations use problem instances from different computational complexity classes, NP-Complete (NPC) and PSPACE-complete (PSC), that exhibit similar solution difficulty but differ in verification difficulty. Our study focuses on nominal groups to isolate the effect of problem complexity on performance. We show that NPC problems have higher demonstrability than PSC problems: participants were significantly more likely to recognize correct and incorrect solutions for NPC problems than for PSC problems. We further show that increasing the group size can actually *decrease* group performance for some problems of low demonstrability. We analytically derive the boundary that distinguishes these problems from others for which group performance monotonically improves with group size. These findings increase our understanding of the mechanisms that underlie group problem-solving processes, and can inform the design of systems and processes that would better facilitate collective decision-making.

## Introduction

A body of social science research has shown the effect of the demonstrability of a problem on a group’s ability to collectively solve intellective problems [1, 2]. A problem is considered to be of high demonstrability if group members who failed to solve the problem are still likely to recognize correct solutions proposed by others. According to the “truth-wins” process [1, 3], when solving problems of high demonstrability, groups are likely to converge to a correct solution as long as there is at least one group member who is able to solve the problem. In contrast, for problems of low demonstrability, members who were not able to correctly solve the problem may not be able to recognize solutions proposed by those who did; thus, the majority of the group might not converge to a correct solution, and the “truth-wins” process does not apply [4].

Prior research also looked at the effect of group size on group performance. Laughlin et al. [4] showed that groups of size three can outperform individual participants in intellective tasks involving arithmetic logic, and Carey and Laughlin [2] demonstrated the superiority of groups over the best individuals when solving coding problems from letters to numbers. Yetton & Bottger [5] showed that the marginal benefit from additional group members reduces with group size for both interacting and nominal groups and that the benefit from additional members depends on their abilities. the conditions under which increasing group size would improve or decrease group performance have not been formalized.

We address this gap by studying the *joint* effect of demonstrability and group size on group performance. We formalize the intuitive notion of demonstrability by drawing on computational complexity theory [6]. Specifically, computational complexity considers two factors: (1) *solution* complexity, how the computational resources required to solve a given problem grow with problem size, and (2) *verification* complexity, how the computational resources required to verify the correctness of a given solution to the problem grows with problem size.

To isolate the effect of the computational complexity of the problem itself on group performance from other aspects that have been shown to affect group performance (e.g., social dynamics in the group), we study nominal (non-interacting) groups. This enables us to understand the computational limitations each individual carries in group interactions. We show through empirical studies and analytical derivations that group performance—and in particular, the effect of group size on performance—depends on both solution and verification complexity. Notably, we show that for problems of particularly low demonstrability, increasing group size can be detrimental to group performance.

We focus on intellective problems with complete information, which require at least some computation and for which there is a ground truth and solutions can be verified for correctness. We distinguish such tasks from judgment tasks where there might not be sufficient information to determine the ground truth during the group’s decision-making process (e.g., a jury’s decision), and quantitative assessment tasks, such as the famous task of assessing the weight of an ox [7], where statistical convergence to the mean makes a larger number of team members beneficial (i.e., the wizdom-of-the-crowd phenomena).

Within the broad category of intellective tasks, we studied two types of problems that are computationally hard, in the sense that the number of possible solutions to consider grows exponentially with problem size. However, they differ in the amount of computation required to verify solutions, hence they should exhibit different levels of demonstrability [8]. The first problem type belongs to the NP-Complete (NPC) computational complexity class, for which solutions can be verified in polynomial time (with respect to the size of the problem). The second problem type belongs to the PSPACE-Complete (PSC) computational complexity class, for which verifying solutions requires exponential time [6]. Amir et al. [8] provided preliminary evidence regarding the relationship between demonstrability and computational complexity. We extend their study in the following ways. First, by showing that for some problems of low demonstrability, groups may fail to converge to the correct solution. Second, by deriving the boundary that distinguishes these problems from others for which group performance monotonically improves with group size. Third, by providing a new empirical design for showing the effects of demonstrability on performance.

We used a nominal group setting in which participants first solved a problem on their own and were then presented with solutions proposed by other group members. We say that a participant is a *solver* (S) of a given problem if the participant was able to solve the problem in a predesignated amount of time (and conversely for a *non-solver* (NS)). A participant has *recognized* a given solution to the problem if the participant was able to accept the solution if it is correct (AC), or reject the solution if it is wrong (RW).

Intuitively, solvers would also be more likely to accept correct solutions (and reject wrong solutions) than non-solvers (i.e., *P*(*AC* ∣ *S*) > *P*(*AC* ∣ *NS*) and *P*(*RW* ∣ *S*) > *P*(*RW* ∣ *NS*)). Therefore, the probabilities of accepting a correct solution and rejecting a wrong solution by a group member (shown in Eq 1) depend on whether the member was able to solve the problem or not, which occurs with probability *P*(*S*) and *P*(*NS*) respectively.

$$\begin{array}{c} \begin{array}{c} P(AC)=P(AC|S)\cdot P(S)+P(AC|NS)\cdot P(NS)\\ P(RW)=P(RW|S)\cdot P(S)+P(RW|NS)\cdot P(NS) \end{array} \end{array}$$ (1)

We define group convergence (GC) as the event by which the majority of the group chooses the correct solution. A necessary and sufficient condition for GC requires that

- A *correct solution exists (ECS) in the set of solutions generated by N group members (at least one of the members was able to solve the problem)*. *P*(*ECS*) = 1 − (1 − *P*(*S*))^*N*^
- *A majority of the group accepts a correct solution*, *given that one exists*, *and a majority rejects wrong solutions*.

$$\begin{array}{c} \begin{array}{cc} P\left(GC|ECS\right)= & \sum_{j>N/2}\left(\begin{array}{c} N\\ j \end{array}\right)P{\left(AC\right)}^{j}\cdot {\left(1-P\left(AC\right)\right)}^{N-j}\cdot \\ & \sum_{j>N/2}\left(\begin{array}{c} N\\ j \end{array}\right)P{\left(RW\right)}^{j}\cdot {\left(1-P\left(RW\right)\right)}^{N-j} \end{array} \end{array}$$ (2)

We posit that NPC problems exhibit high demonstrability due to their easy-to-verify nature (and conversely for PSC problems). Accordingly, we formulate the following hypotheses:

**Hypothesis 1**: The probability of a non-solver accepting a correct solution (and rejecting a wrong solution) will be higher for NPC problems than for PSC problems:

$$\begin{array}{c} P_{NPC}\left(AC|NS\right)>P_{PSC}\left(AC|NS\right),P_{NPC}\left(RW|NS\right)>P_{PSC}\left(RW|NS\right) \end{array}$$ (3)

**Hypothesis 2**: Increasing the group size for NPC problems will improve group performance (i.e., facilitate convergence to the correct solution) because non-solvers will be able to recognize a correct solution when it is presented to them.

$${\text{lim}}_{n\to \infty }P_{NPC}\left(GC\right)\to 1$$ (4)

(Note that the term *P*(*ECS*) converges to 1 for both problem classes so we can replace *P*(*GC* ∣ *ECS*) with *P*(*GC*).)

In contrast, increasing group size for PSC problems may be *detrimental* to group performance, because non-solvers might not recognize the correct solution. That is, for at least some PSC type problems, we expect the following to hold:

$$\begin{array}{c} lim_{n\to \infty }P_{PSC}\left(GC\right)\to 0 \end{array}$$ (5)

**Fig 1:**
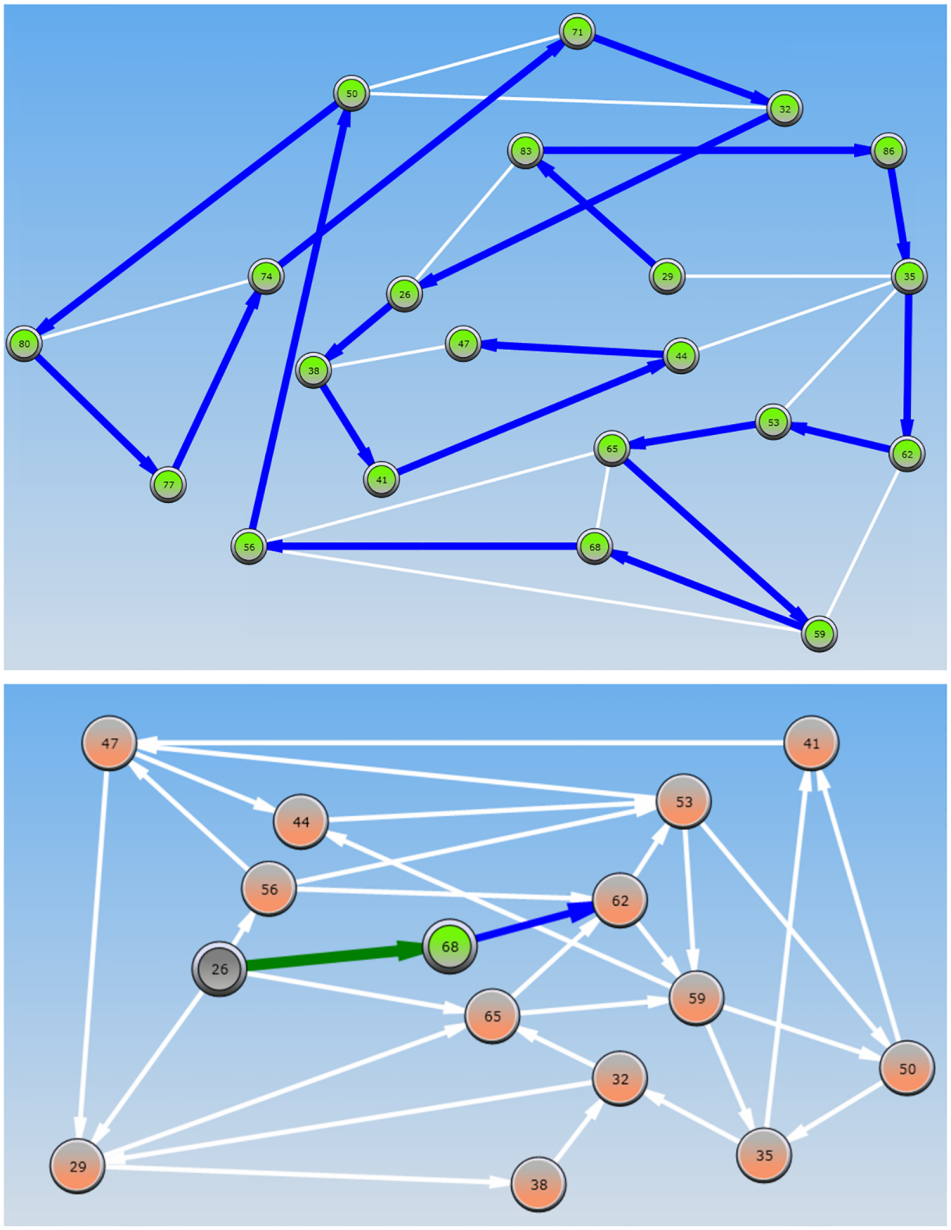
Screen-shots of the TSP-H (top) and GEO-H (bottom) problem instances.

For the NPC class, we used the traveling salesman problem (TSP), which requires the solver to form a closed loop through the graph that visits each node exactly once. For the PSC complexity class, we used a strategic game called Geography (GEO), in which players traverse a path on the graph by selecting a node at each turn, starting from an initial node. The first player to reach a node which does not have outgoing edges, or only has outgoing edges to nodes that were previously chosen (a.k.a “sink”) loses the game. For both problems, we generated an easy instance (denoted TSP-E and GEO-E respectively) and a hard instance (denoted TSP-H and GEO-H respectively). Fig 1 (top) shows a visualization of a possible solution to the TSP-H problem with a solution emanating from the node labeled 29 and terminating with the node labeled 47 having traversed the entire graph with no cycles. Fig 1 (bottom) shows the GEO-H problem instance in which the green player is positioned at node 26 and is asked to choose the next node which will guarantee a win over the blue player.

The Institutional Review Board (IRB) of the Department of Software and Information Systems Engineering at Ben-Gurion University approved the described experiments. All the participants provided their informed consent to participate in the study.

Participants in online experiments (*N* = 296) were assigned to one of four conditions, varying the type of the problem (TSP or GEO) and the difficulty of solving the problem (Hard or Easy). Participants first solved the problem individually and submitted their solutions. They were then presented with three possible solutions to the problem: a correct solution, a wrong solution, and their own solution. For each of the solutions, participants were asked to accept the solution as correct, or reject the solution as incorrect.

## Results

Table 1 shows the number of participants and the proportion of participants (*P*(*S*)) who solved the hard problems TSP-H and GEO-H. These two problems exhibited similar *P*(*S*) values of 0.173 and 0.188 respectively (*χ*^2^(1) = 0.00036, *p* = 0.9848). Thus, the empirical difficulty of *solving* the two types of problems was similar. There was also no significant difference in the empirical difficulty of solving the easy problems TSP-E and GEO-E (see Fig B in S1 File).

**Table 1:** Number of subjects and *P*(*S*) measures for GEO-H and TSP-H problems.

	TSP-H	GEO-H
num.	85	69
*P*(*S*)	0.18	0.17

**Fig 2:**
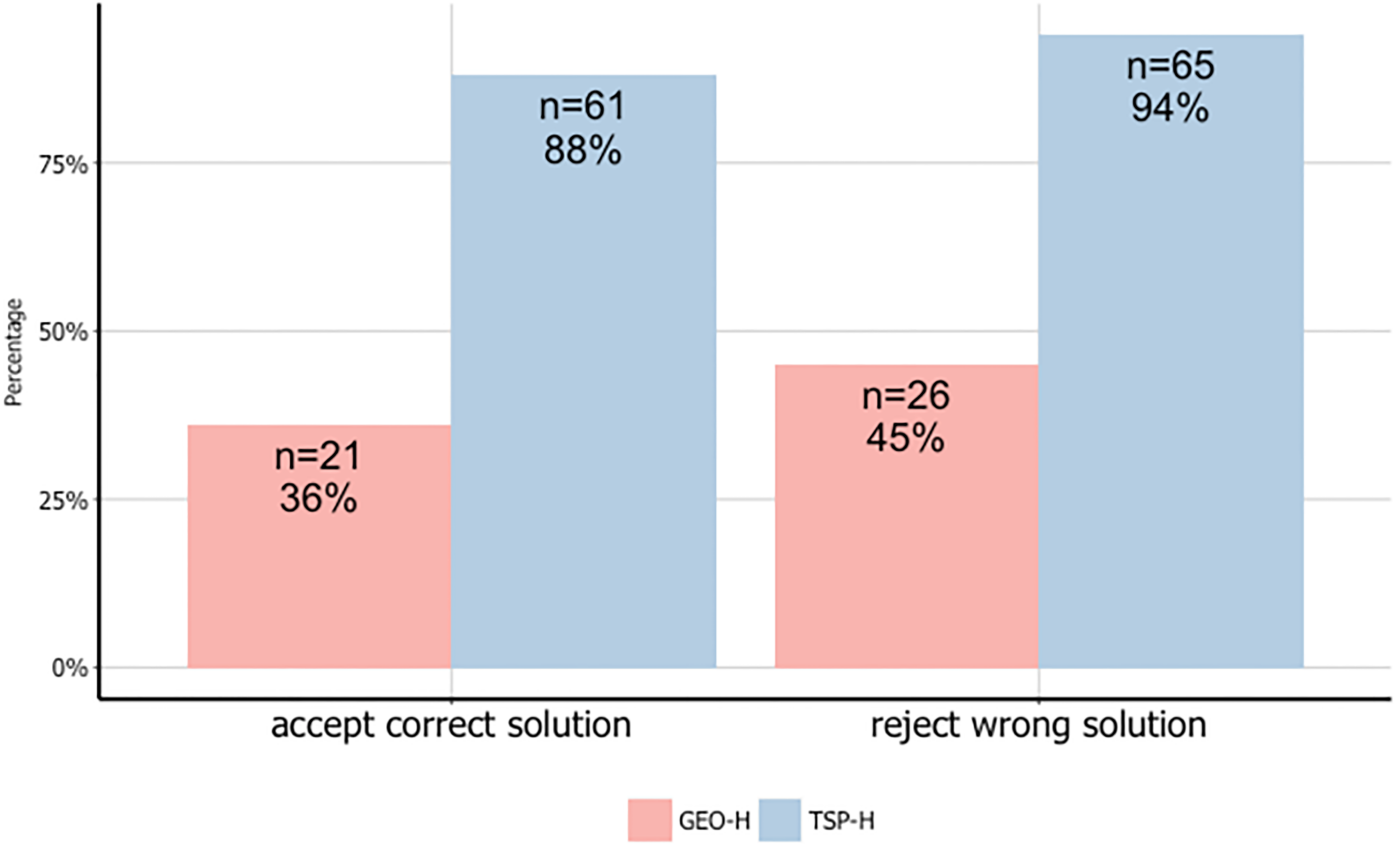
Non-Solvers’ acceptance and rejection of solutions for TSP-H (an NPC-type problem) and for GEO-H (a PSC-type problem).

Fig 2 shows non-solvers’ ability to recognize solutions for TSP-H and GEO-H problems. As shown in the figure, 88% of non-solvers (out of 69) accepted a correct solution for the TSP-H problem, compared to 36% (out of 57) for the GEO-H problem. Similarly, 94% of non-solvers rejected a wrong solution for the TSP-H problem, compared to 45% for the GEO-H problem. All differences were statistically significant (*χ*^2^(1) > 34.285, *p* < 0.001). The results for the TSP-E and GEO-E problems were similar, supporting Hypothesis 1. We validated our results by repeating the experiment in laboratory settings (N = 55) (see Tables C and D in S1 File.)

**Fig 3:**
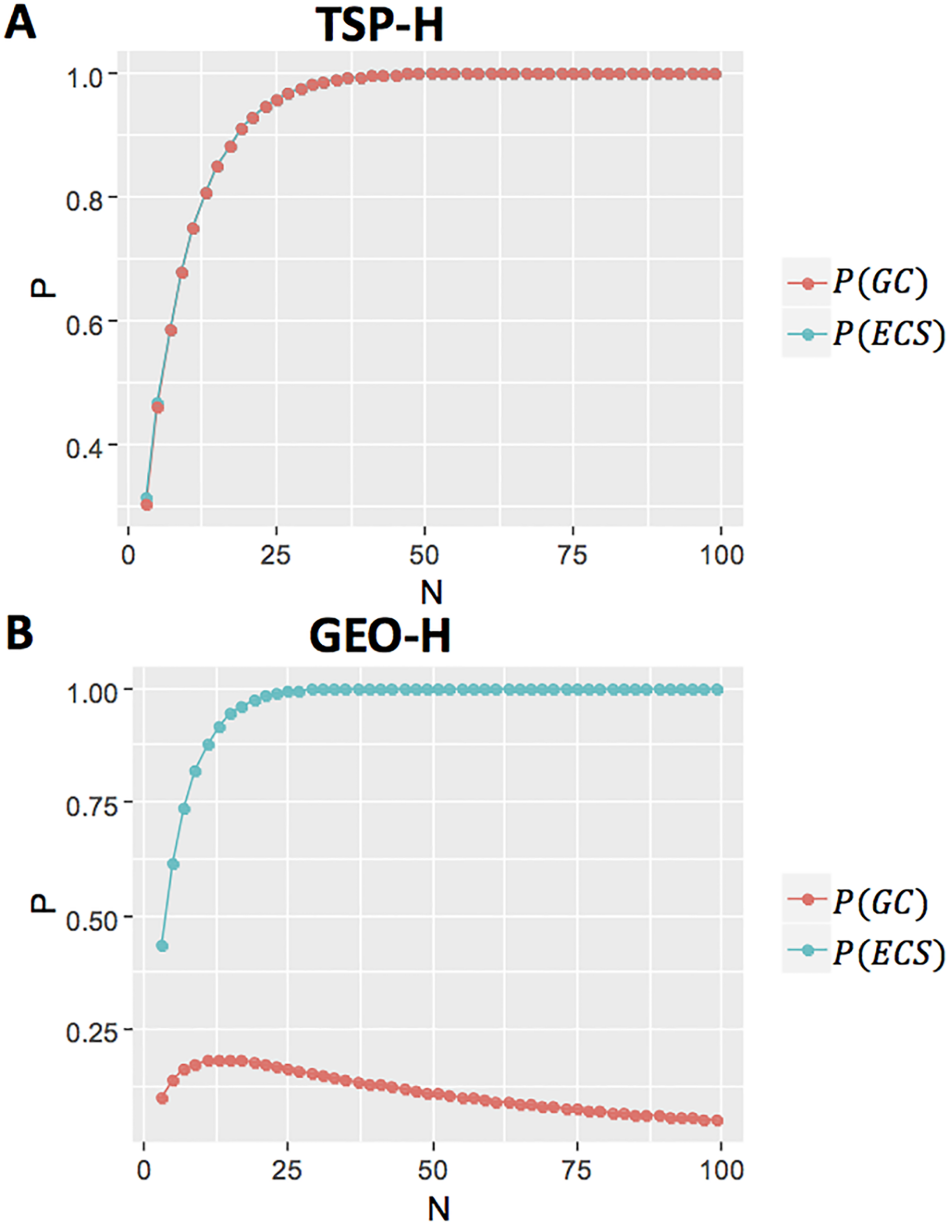
The probability of the group converging to the correct solution, *P*(*GC*) (red curve), and of at least one group member solving the problem, *P*(*ECS*) (blue curve), given the number of group members, for TSP-H problem (an NPC-type problem) and for the GEO-H problem (a PSC-type problem). For TSP-H, *P*(*GC*) monotonically increases as *P*(*ECS*) increases, ultimately converging to 1. In contrast, for GEO-H, *P*(*GC*) initially increases (due to the increase in *P*(*ECS*)), but reaches a peak at *N* = 12 and then decreases as a result of participants’ inability to correctly verify solutions.

To test Hypothesis 2, we ran simulations of group performance (*GC*) for different group sizes. We used the experimentally obtained values (*P*(*S*), *P*(*AC*), *P*(*RW*)) for the TSP-H problem (Fig 3(A)) and GEO-H problem (Fig 3(B)). For both problems, *P*(*ECS*) (the likelihood that a solution is generated by at least one participant, shown by the blue line) quickly increases with group size. However, for the TSP-H problem, *P*(*GC*) (group performance, shown by the red line) converges with *P*(*ECS*), whereas for the GEO-H problem, despite the increase of *P*(*ECS*) with group size, the group performance decays when *N* > 12 due to the inability of group members to recognize correct solutions (Fig 3(B)). These results support Hypothesis 2.

The empirical results and simulations supporting Hypothesis 2 show that for problems of particularly low demonstrability (such as hard PSC problems), increasing group size beyond a certain finite number is detrimental to group performance. Intuitively, the reason for this detrimental effect is that the benefit of adding group members is marginally decreasing, because at some point the likelihood of having at least one group member who correctly solves the problem converges to 1, and beyond this point adding more group members is no longer beneficial. At the same time, increasing the group size monotonically *decreases* the likelihood that a majority of group members will accept the correct solution and reject the wrong solutions. Therefore, beyond a certain optimal finite group size, this negative effect will outweigh the positive effect of increasing the likelihood of generating a correct solution.

Next, we generalize this result by characterizing the phase transition between two types of problems: problems for which increasing group size monotonically improves performance, and problems for which performance peaks at a finite optimal group size and decays thereafter.

In this analysis, we assume for simplicity that solvers can always correctly verify other wrong/correct solutions (i.e., *P*(*AC* ∣ *S*) = *P*(*RW* ∣ *S*) = 1)) and that for a non-solver, the likelihoods of verifying a correct solution and verifying a wrong solution are equal (i.e., *P*(*AC* ∣ *NS*) = *P*(*RW* ∣ *NS*)). We can now use a single term, *P*(*VC*), to denote the likelihood that a given solution was verified correctly as right or wrong (where *P*(*VC*) = *P*(*AC*) = *P*(*RW*)).

### Numerical simulation and separatrix boundary

**Fig 4:**
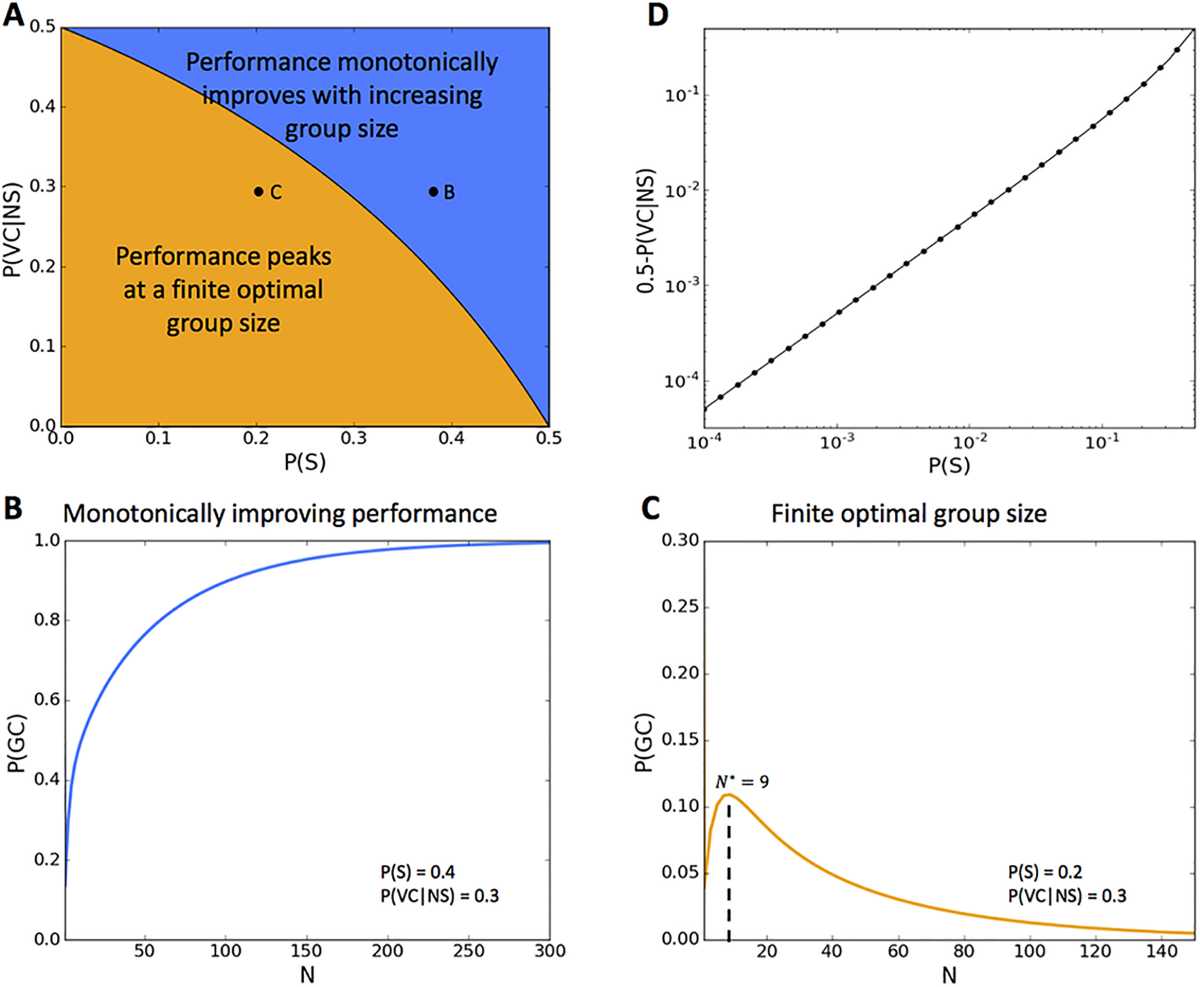
(A) Numerical simulations show two phases of performance depending on group size. The first phase describes groups whose performance increases monotonically with the number of group members (blue). The second phase (orange) describes groups whose performance reaches a maximum at a finite group size and decays thereafter. (B) Group performance (*P*(*GC*)) increases monotonically with increasing group size (*P*(*S*) = 0.4, *P*(*VC* ∣ *NS*) = 0.3). *P*(*GC*) approaches 1 as *N* → ∞; (C) Group performance (*P*(*GC*)) reaches a peak at a finite group size (*N* = 9) and decays for increasing group size, for a particular set of problem parameters (*P*(*S*) = 0.1, *P*(*VC* ∣ *NS*) = 0.4). *P*(*GC*) approaches 0 as *N* → ∞. (D) The separatrix between the two phases follows a simple analytic relation: $P\left(VC|NS\right)=\frac{0.5-P(S)}{1-P(S)}$. The points on the separatrix line are computed by simulation and align with this analysis.

Using numerical simulations for different parameter values of *P*(*S*) and *P*(*VC* ∣ *NS*), we found two different phases of group behavior, a region where performance improves monotonically with group size (Fig 4(A), blue region) and a region where increasing group size decreases performance after a peak at a finite group size (Fig 4(A), orange region). Fig 4(B) and Fig 4(C) show the group performance for example problem instances that lie in the different regions.

We derive the separatrix between the two phases analytically. We model group convergence as a binomial process, where each group member succeeds in recognizing the correct solution with a probability of *P*(*VC*) (similar to the process described by Eq 2). Adding a group member results in an additional trial to this process. If *P*(*VC*) > 0.5, the additional group member is *more* likely to verify solutions correctly than not, thus *increasing* the overall likelihood that the group will converge to the correct solution. If, however, *P*(*VC*) < 0.5, the additional group member is *less* likely to verify solutions correctly than not, and therefore adding this group member *decreases* the overall likelihood that the group will converge to the correct solution. This process is analogous to the tossing a biased coin: if the coin is biased in favor of getting “heads”, increasing the number of coin tosses increases the likelihood of obtaining a majority of “heads”. We can therefore compute for each *P*(*S*) the minimal value of *P*(*VC*) such that increasing *N* will always increase *P*(*GC* ∣ *ECS*), by requiring that *P*(*VC*) > 0.5. To obtain *P*(*VC*) > 0.5, we have

$$\begin{array}{c} \begin{array}{cc} & 1\cdot P(S)+P(VC|NS)\cdot (1-P(S))>0.5\\ & P\left(VC|NS\right)>\frac{0.5-P(S)}{1-P(S)} \end{array} \end{array}$$ (6)

Fig 4(D) shows the separatrix boundary on a log-log scale based on the analytical derivation of Eq 6. The points on the separatrix boundary in the figure are computed by simulation and coincide with the equation. The problems from our empirical problems fall in different regions of this space. The NPC type problems (TSP-H, TSP-E) fall within region B (in blue) whereas the PSC type problems (Geo-H, GEO-E) fall within region C (in orange). The Supplementary Information also includes analysis of the optimal group size for problems within this region (Fig B in S1 File).

## Discussion and conclusion

The results of our study contribute to the literature on group decision making regarding strategic/combinatorial problems. They relate to the ongoing debate as to when groups are better than individuals, and demonstrate that the nature of the problem to be solved might be as important as the characteristics of the group members attempting to solve it. Groups have been shown to outperform individuals in lab settings [4, 9, 10] and in certain prediction and estimation tasks, typically involving a relatively straightforward quantitative judgment (the “wisdom of the crowd” effect [7, 11, 12]). In another study of quantitative judgment tasks, it has been shown that the improved performance of groups can sometimes be attributed to learning that occurs in individuals as a result of group discussions [13]. Our results show that large groups might not be preferable when solving more complex problems which are characterized by low demonstrability. On the other hand, many studies have documented that certain dynamics (polarization, free-riding and “groupthink”) may seriously inhibit the group’s overall performance [14–20], and that coordination costs further inhibit performance as the team gets larger [21].

Our work complements these previous studies which describe deleterious effects of small group dynamics. Our results underline the important role of problem complexity in group processes, even before considering the different personal dynamics such a group may display. We show that in addition to potential detrimental effects of social dynamics on group performance, there are also detrimental effects stemming from the difficulty individuals have in assessing the correctness of proposed solutions.

Regarding the study’s limitations, we have based our model and empirical studies on non-interacting (nominal) groups, which is distinct from situations in which groups solve problems together [9, 22–24]. Studying nominal groups enabled us to focus on the inherent characteristics of the *problem* and their effects on group performance, eliminating factors related to group dynamics. We note that such groups are becoming prevalent in online communities such as crowdsourcing platforms and citizen science [25, 26].

Second, in our study each participant was presented with a single wrong solution. In general, there could be many wrong solutions, and our formula *P*(*RW*) can easily be extended by adding similar elements for each of these wrong options. Having multiple wrong solutions will actually make group convergence more difficult for PSC problem types, because solvers can be led astray by more options. In this respect, the likelihood of convergence for PSC problems shown in Fig 4, when there is just one wrong solution, represents an upper bound.

To summarize, our results show that the benefit from increasing the group size (*P*(*ECS*) increases with *N*) can be offset by the fact that its members may not recognize correct solutions (*P*(*VC*) is low). One possibility for mitigating the detrimental effect of increasing the group size, due to the inability of group members to identify correct solutions, is to separate the group that generates solutions from a group of experts that choose the best solutions. This design choice is exhibited in an open innovation platform that uses a group of experts to choose the winning ideas posed by the crowd [27].

As the world becomes more connected, groups are increasingly able to solve problems collaboratively by utilizing participants of diverse backgrounds and expertise [28–30]. This study improves our understanding of the mechanisms that underlie group problem-solving processes, and can inform the design of systems for helping groups make good decisions collectively.

## Supporting information

S1 File Supplementary Figs and Tables. **Table A**, Solvers’ acceptance and rejection of solutions for TSP-E, GEO-E, GEO-H and TSP-H. **Table B**, Average verification time and standard deviation (in parentheses) in seconds for each problem instance. **Table C**, Number of subjects from Ben-Gurion and *P*(*S*) measures (no interaction groups). **Table D**, Ben-Gurion Solvers’ acceptance and rejection of solutions for GEO-H and TSP-H. **Fig A**, Screen shots of easy instances for traveling sales-person (top) and Geography (bottom) problems. **Fig B**, Non-Solvers’ acceptance and rejection of solutions for TSP-E and GEO-E. **Fig C**, Non-Solvers’ acceptance and rejection of solutions for TSP-H and GEO-H. **Fig D**, The optimal group size (N*) scales inversely to the difficulty of the problem (*P*(*S*)). The relationship between N* and 1/*P*(*S*) is shown on a log-log scale.
(PDF)

S2 File Lab consent form. Consent form informed consent to participate in the online study.
(PDF)

S3 File Instructions for subjects. Instructions for subjects in the GEO condition.
(PDF)

## References

[1] Laughlin PR, Ellis AL. Demonstrability and social combination processes on mathematical intellective tasks. Journal of Experimental Social Psychology. 1986;22(3):177–189. doi: 10.1016/0022-1031(86)90022-3

[2] Carey HR, Laughlin PR. Groups perform better than the best individuals on letters-to-numbers problems: Effects of induced strategies. Group Processes & Intergroup Relations 2011;.

[3] Lorge I, Solomon H. Two models of group behavior in the solution of eureka-type problems. Psychometrika. 1955;20(2):139–148. doi: 10.1007/BF02288986

[4] Laughlin P, Hatch E, Silver J, Boh L. Groups Perform Better Than the Best Individuals on Letter-to-Numbers Problems: Effects of Group Size. Journal of Personality and Social Psychology. 2006;4(4):644–651. doi: 10.1037/0022-3514.90.4.644 10.1037/0022-3514.90.4.644 16649860

[5] Yetton P, Bottger P. The relationships among group size, member ability, social decision schemes, and performance. Organizational Behavior and Human Performance. 1983;32(2):145–159. doi: 10.1016/0030-5073(83)90144-7

[6] Johnson D. Computers and Intractability-A Guide to the Theory of NP-Completeness; 1979.

[7] Surowiecki J. The wisdom of crowds. Anchor; 2005.

[8] Amir O, Shahar Y, Gal Y, Ilani L. On the verification complexity of group decision-making tasks. In: First AAAI Conference on Human Computation and Crowdsourcing; 2013.

[9] Bradley BH, Baur JE, Banford CG, Postlethwaite BE. Team Players and Collective Performance: How Agreeableness Affects Team Performance Over Time. Small Group Research. 2013;44(6):680–711. doi: 10.1177/1046496413507609

[10] Hill GW. Group versus individual performance: Are N+ 1 heads better than one? Psychological Bulletin. 1982;91(3):517 doi: 10.1037/0033-2909.91.3.517

[11] Malone TW, Laubacher R, Dellarocas C. Harnessing crowds: Mapping the genome of collective intelligence. MIT; 2009.

[12] Yi SKM, Steyvers M, Lee MD, Dry MJ. The wisdom of the crowd in combinatorial problems. Cognitive science. 2012;36(3):452–470. doi: 10.1111/j.1551-6709.2011.01223.x 22268680 10.1111/j.1551-6709.2011.01223.x

[13] Schultze T, Mojzisch A, Schulz-Hardt S. Why groups perform better than individuals at quantitative judgment tasks: Group-to-individual transfer as an alternative to differential weighting. Organizational Behavior and Human Decision Processes. 2012;118(1):24–36. doi: 10.1016/j.obhdp.2011.12.006

[14] Callaway MR, Marriott RG, Esser JK. Effects of dominance on group decision making: Toward a stress-reduction explanation of groupthink. Journal of Personality and Social Psychology. 1985;49(4):949 doi: 10.1037/0022-3514.49.4.949 4057051 10.1037//0022-3514.49.4.949

[15] Kim Y. A comparative study of the “Abilene Paradox” and “Groupthink”. Public Administration Quarterly 2001; p. 168–189.

[16] Harvey JB. The Abilene paradox: The management of agreement. Organizational Dynamics. 1974;3(1):63–80. doi: 10.1016/0090-2616(74)90005-9

[17] Koriat A. When Are Two Heads Better than One and Why? SCIENCE. 2012;336(April):360–362. doi: 10.1126/science.1216549 22517862 10.1126/science.1216549

[18] Dessein W. Why a Group Needs a Leader: Decision-making and Debate in Committees. C.E.P.R. Discussion Papers; 2007. 6168. Available from: https://ideas.repec.org/p/cpr/ceprdp/6168.html.

[19] Sapir L. The optimality of the expert and majority rules under exponentially distributed competence. Theory and Decision. 1998;45(1):19–36. doi: 10.1023/A:1005094032398

[20] Visser B, Swank OH. On committees of experts. Quarterly Journal of Economics. 2007;122(1):337–372. doi: 10.1162/qjec.122.1.337

[21] Hackman JR. Why teams don’t work In: Theory and research on small groups. Springer; 2002 p. 245–267.

[22] Anita Williams W, Christopher F C, Alexander P, Nada H, Thomas W M. Evidence for a Collective Intelligence Factor in the Performance of Human Groups. SCIENCE. 2010;330(29 October):686. 20929725 10.1126/science.1193147

[23] Dong W, Lepri B, Pentland A. Automatic prediction of small group performance in information sharing tasks. Collective Intelligence 2012;.

[24] Campbell J. The Influence of Time and Task Demonstrability on Decision-Making in Computer-Mediated and Face-to-Face Groups. Small Group Research. 2006;37(3):271–294. doi: 10.1177/1046496406288976

[25] Bonney R, Cooper CB, Dickinson J, Kelling S, Phillips T, Rosenberg KV, et al Citizen science: a developing tool for expanding science knowledge and scientific literacy. BioScience. 2009;59(11):977–984. doi: 10.1525/bio.2009.59.11.9

[26] Kittur A, Nickerson JV, Bernstein M, Gerber E, Shaw A, Zimmerman J, et al. The future of crowd work. In: Proceedings of the 2013 conference on Computer supported cooperative work. ACM; 2013. p. 1301–1318.

[27] Lakhani KR, Fayard AL, Levina N, Pokrywa SH. OpenIDEO. Social Science Research Network (SSRN); 2012.

[28] Boudreau KJ, Lakhani KR. Using the crowd as an innovation partner. Harvard business review. 2013;91(4):60–69. 23593768

[29] Chesbrough HW. Open innovation: The new imperative for creating and profiting from technology. Harvard Business Press; 2006.

[30] Chesbrough HW. The era of open innovation. Managing innovation and change. 2006;127(3):34–41.

