# Supplementary material for: The more the merrier? Increasing group size may be detrimental to decision-making performance in nominal groups
Source: PLoS One. 2018 Feb 27;13(2):e0192213. doi: 10.1371/journal.pone.0192213 (PMC5828441; doi:10.1371/journal.pone.0192213)
Supplement: S1 File — Table A, Solvers’ acceptance and rejection of solutions for TSP-E, GEO-E, GEO-H and TSP-H. Table B, Average verification time and standard deviation (in parentheses) in seconds for each problem instance. Table C, Number of subjects from Ben-Gurion and P(S) measures (no interaction groups). Table D, Ben-Gurion Solvers’ acceptance and rejection of solutions for GEO-H and TSP-H. Fig A, Screen shots of easy instances for traveling sales-person (top) and Geography (bottom) problems. Fig B, Non-Solvers’ acceptance and rejection of solutions for TSP-E and GEO-E. Fig C, Non-Solvers’ acceptance and rejection of solutions for TSP-H and GEO-H. Fig D, The optimal group size (N*) scales inversely to the difficulty of the problem (P(S)). The relationship between N* and 1/P(S) is shown on a log-log scale. (PDF) [file pone.0192213.s001.pdf]

The More the Merrier? Increasing Group Size may be Detrimental to Decision-Making Performance in Nominal Groups

Ofra Amir[1], Dor Amir[2], Yuval Shahar[2], Yuval Hart[3], and Kobi Gal[2]

[1]Technion - Israel Institute of Technology, Israel
[2]Ben-Gurion University, Israel
[3]Harvard University, USA

1 Additional Information About Empirical Study

1.1 Empirical Methodology

The empirical results reported in the paper were based on studies in which participants were recruited thorough Amazon Mechanical Turk (MTurk). The results for some of the conditions were replicated in lab studies conducted in Ben-Gurion University. In this section, we provide additional details about task design and analyses for all experiments.

1.1.1 Task

We generated two instances of each problem type, the Geography game (GEO), which belongs to the PSC computational complexity class, and Traveling Salesperson (TSP), which belongs to the NPC computational complexity class. For TSP, the easier problem instance (TSP-E) consisted of 15 nodes and 22 edges. The harder problem instance (TSP-H) consisted of 20 nodes and 31 edges. For GEO, the easier problem instance (GEO-E) consisted of 20 nodes and 30 edges, with three outgoing edges emanating from the initial starting vertex. The harder GEO instance (GEO-H) consisted of 15 nodes and 31 edges.

We developed a graphical user interface for visualizing both problem types and for providing support for participants when reasoning about the problem instances. Nodes were labeled with numbers and participants traverse the graph by selecting adjacent nodes on the screen, which activates the edge between adjacent nodes. Figure A (top) shows a visualization of the possible solution to the TSP-E problem with a solution emanating from the node labeled 56 and terminating with the node labeled 38 having traversed the entire graph with no cycles. This TSP-E instance includes 15 nodes and 22 edges, while the TSP-H instance included 21 nodes and 31 edges. For

GEO games, participants were assigned an initial position on the graph for the first player (Green), and could simulate moves for themselves and for the other player (Blue) by selecting nodes on the graph. The problem instances were designed such that (assuming Blue plays optimally) the Green player could force a win by choosing exactly one of the nodes emanating from its starting position, while all other nodes lead to a win of Blue. There were six emanating edges from the starting position of the GEO-E problem instance and four emanating edges from the starting position of the GEO-H problem instance. Nodes were shown in green for player 1s turns and in blue for player 2s turns. Figure A (bottom) shows the visualization of the GEO-E problem instance from the point of view of the Green player. The initial node label in this problem was 26. The Green player chose the node labeled 53 (the other options were nodes labeled 29, 56, 65, 71 and 77. Then the Blue player chose the node labeled 32 as this is the only node possible given the path. For both TSP and GEO visualizations, participants were provided with a "submit" button to submit their solution. They were also provided with an "undo" button for reversing the previous action, and a reset button for removing their work thus far and refreshing the problem instance display.

1.1.2 Participants

MTurk participants. We recruited 296 subjects through Amazon Mechanical Turk. We restricted participation to participants from the US. We used a between-subject design where each participant was assigned to one of four conditions: TSP-E, GEO-E, GEO-H and TSP-H. The GEO-E condition included 51 participants (16 females) between the age of 19 to 60 ($mean = 31.5$), while 69 participants (25 females) between the ages of 18 to 65 ($mean = 31.5$) were assigned to the GEO-H condition. Ninety one participants (30 females) between the age of of 19 to 65 ($mean = 31.5$)were assigned to the TSP-E condition. Eighty five participants (33 females), between the ages of 18 to 60 ($mean = 31.6$) were assigned to the TSP-H condition. We found no statistical difference between the behavior of males and females in the study. Most of the participants (187 out of 296) were college students.

Lab participants. We recruited 55 undergraduate students from the faculty of engineering sciences at Ben-Gurion University. Each participant completed both the TSP-H or GEO-H conditions (within-subject design).

1.1.3 Procedure

MTurk study procedure. After answering a short demographic questionnaire, participants were presented with a tutorial detailing their respective problem and the appropriate use of the GUI[1]. To verify that participants understood the problem and the use of the GUI, participation was contingent on successfully passing a quiz regarding the task, which included questions regarding a simple instance of the problem. Participants were presented with one of the problem instances based on their assigned condition and were asked to solve it individually. We restricted solution time to 5 minutes. Upon submitting their answers, participants were asked to explain how they solved the problem, and why their solution is correct. Finally, participants were presented with three possible

[1]lab participants were provided a Hebrew translation of the tutorial presented to MTurk participants.

solutions to the problem they solved: the participants own solution, a pre-generated correct solution to the problem and a pre- generated incorrect solution to the problem. These pre-generated solutions represented common mistakes that were submitted by participants. The participant's own solution was always presented last, to avoid priming the subject. The order of the pre-generated correct and incorrect solutions were randomized.

Participants were compensated based on their performance. they received a base payment of \$0.7 and could earn a bonus of up to \$1.4 based on their performance: they received a bonus of \$0.35 for correctly solving the problem and an additional bonus of \$0.35 for each solution they were able to correctly classify as correct or incorrect.

Lab study procedure. For the lab study we used a within-subject design which included only the GEO-H and TSP-H conditions. For each of the two problem instances, the procedure was identical to the one used in the MTurk study. Participants received 1 bonus point in a course for their participation and also received monatery compensation based on their performance: 5 NIS (\$1.3) for solving the problem correctly and additional 5 NIS for correctly verifying the solutions presented to them.

Analyses. We wished to exclude participants who guessed solutions to the problem from our analyses. Therefore, we excluded from our analyses participants who had fewer than 10 interactions with the GUI, measured by the number of times they selected nodes on the interface, or completed the problem solving session in less than 2 minutes. These criteria were determined based on a pilot study. We excluded participants who did not pass these thresholds from the MTurk study, resulting in a total of $N = 296$ participants in our analysis. We did not exclude any lab participants.

Our main hypothesis suggests that PSC and NPC problems will exhibit different likelihoods of verifying solutions correctly. Therefore, our analysis compares the proportions of participants who were able to accept correct solution ($P(AC)$) and reject wrong solutions ($P(RW)$). We used the z-test for proportions to determine the significance of differences. We also use the proportion test to compare the likelihoods of solving and verifying solutions observed in the MTurk and lab studies. Last, we compare the verification times for the different problems. For this analysis, we use a t-test to determine statistical significance.

1.2 Additional Results

In this section we provide additional results beyond those described in the main menuscript. Specifically, for the MTurk experiments, we describe the results for the easy problem instances and an analysis of verification times. We then report the results of the lab study for the hard instances, which are inline with the MTurk study results and provide further evidence supporting our hypotheses.

1.2.1 Results for Easy Problem Instances

Fig. B presents the results for the easy-to-verify problems.

The results for the easy instances are similar to those obtained for the hard instances, and provide additional support for **Hypothesis 1**. As can be seen in the table, despite not being able to solve the problem on their own, most *non-solvers* in the TSP-E condition were able to correctly

reject wrong solutions and accept correct solutions. Only 2 wrong solutions of 39 (0.051) were accepted by participants, and only 7 of 39 correct solutions were rejected (0.179). In contrast, wrong solutions were more often accepted by non-solvers in the GEO-E condition (0.37) and similarly a significantly higher proportion of correct solutions were rejected (0.629). The differences in proportions were statistically significant for both $P(AC)$ and $P(RW)$ ($\chi^2 > 12.093, p < 0.0005$).

While our analysis focuses on non-solvers, the accuracy of *solvers* in classifying other solutions, shown in Table A, suggests that even for solvers, classifying other solutions was harder in the PSC condition (likelihood of 0.167 of accepting wrong solutions in GEO-E compared to 0 in TSP-E). Thus, in addition to non-solvers having a hard time in recognizing which of the solutions is correct, even solvers of PSC problems might be swayed towards a wrong solution, further reducing the likelihood of convergence to a correct solution.

	TSP-E		GEO-E		TSP-H		GEO-H	
	Correct	Wrong	Correct	Wrong	Correct	Wrong	Correct	Wrong
Accept	47	0	22	4	16	0	0	3
Reject	5	52	2	20	0	16	4	26

Table A: Solvers' acceptance and rejection of solutions for TSP-E, GEO-E, GEO-H and TSP-H

1.2.2 Verification Times

We examined the time participants spent verifying solutions, as these times provide another indication to the difficulty of determining whether a presented solution is correct or not. Table B shows the average time for each problem and each solution type (correct, incorrect or the participant's own solution). We observe that participants spent significantly less time verifying correct and incorrect solutions to the TSP-E problem, compared to the time they spent verifying solutions to GEO-E. All differences are statistically significant ($t(140) > 3.51, p < 0.001$). We observe similar results when comparing verification times for TSP-H and GEO-H, with even greater differences between the two problem types ($t(152) > 4.585, p < 1e-05$).

Condition	Correct Solution	Incorrect Solution	Own Solution
GEO-E	35.79 (29.01)	44.51 (32.13)	26.73 (32.84)
TSP-E	22.76 (15.27)	13.70 (9.76)	19.95 (16.53)
GEO-H	57.63 (62.75)	50.78 (57.66)	40.03 (88.39)
TSP-H	24.82 (16.90)	15.31 (12.68)	12.86 (8.67)

Table B: Average verification time and standard deviation (in parantheses) in seconds for each problem instance.

1.2.3 Results of Lab Experiment

Table C shows the number of students in each problem type (TSP-H and GEO-H). We did not find a statistically significant difference between $P(S)$ in studies conducted in the lab and Mturk (z-test

	TSP-H	GEO-H
num.	55	55
P(S)	0.18	0.25

Table C: Number of subjects from Ben-Gurion and $P(S)$ measures (no interaction groups)

	TSP-H		GEO-H	
	Correct	Wrong	Correct	Wrong
Accept	39	1	19	25
Reject	2	40	26	20

Table D: Ben-Gurion Solvers' acceptance and rejection of solutions for GEO-H and TSP-H

$p = 1$).

Figure C shows the number of non-solver students in each condition that accepted a solution as correct, or rejected the solution as incorrect. For both correct and incorrect solutions, there was no statistically significant difference between the acceptance rate in the lab and Mturk studies (z-test, $p = 0.7$ for correct solutions and $p = 0.4229$ for incorrect solutions for TSP-H problem; $p = 0.5791$ for correct solutions and $p = 0.9037$ for incorrect solutions for GEO-H problems).

Table D shows the number of solver students in each condition that accepted a solution as correct, or rejected the solution as incorrect. For both correct and incorrect solutions, there was no statistically significant difference between the acceptance rate between the lab and Mturk ($z-test$, For TSP-H $p = 0.2749$ for correct solutions and $p = 0.1174$ for incorrect solution and for GEO-H $p = 0.1925$ for correct solutions and $p = 0.7805$ for incorrect solutions).

2 Additional Analysis of Separatrix Boundary

The analysis of the separatrix boundary described in the paper assumed $P(AC) = P(RW)$. It can be generalized to the case that $P(AC) \neq P(RW)$ by considering the multiplication of two binomial processes, one determining whether a majority of the group members were able to accept the correct solution, and similarly one for determining whether a majority of the group members were able to reject wrong solutions. Therefore, a sufficient condition for ensuring that the group would monotonically improve when increasing group size is to have both $P(AC) > 0.5$ and $P(RW) > 0.5$.

The relationship between the optimal group size and problem difficulty. For problems that lie in the region of finite optimal group size (low demonstrability), the value of the optimal group size N^* depends on the difficulty of solving the problem ($P(S)$). Recall that for such problems, while increasing N increases the likelihood that some group member would generate a correct solution (greater $P(ECS)$), it also reduces the likelihood that a majority of the group would be able to correctly verify solutions ($P(VC) < 0.5$). Therefore, adding group members beyond some point will be detrimental to group performance.

Intuitively, the harder it is to solve a problem (lower $P(S)$), the greater the number of group members needed to ensure that at least one correct solution is needed. Therefore, we expect the value of N^* to be higher for problems with lower values of $P(S)$. We ran simulations to determine the relationship between N^* and $P(S)$. Figure D shows the results for problems that lie close to the separatrix boundary ($P(VC) = 0.5 - \epsilon$, where $\epsilon = 10^{-6}$). As can be seen in the figure, N^* scales with $1/P(S)$. For smaller values of $P(VC)$, the optimal group size is smaller, as the harmful effect of adding group members on verification is more substantial. However, for a given value of ϵ, the value of N^* scales with $1/P(S)$, demonstrating the same trend as shown in Figure D.

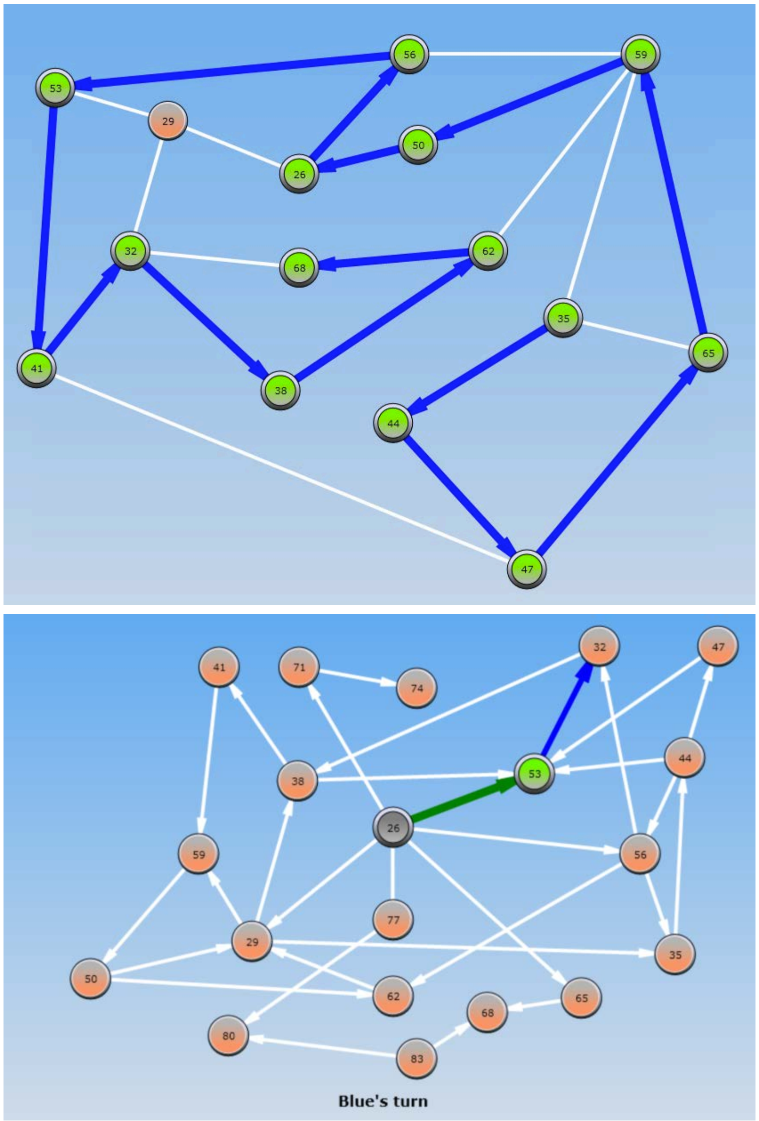

Figure A: Screen shots of easy instances for traveling sales-person (top) and Geography (bottom) problems.

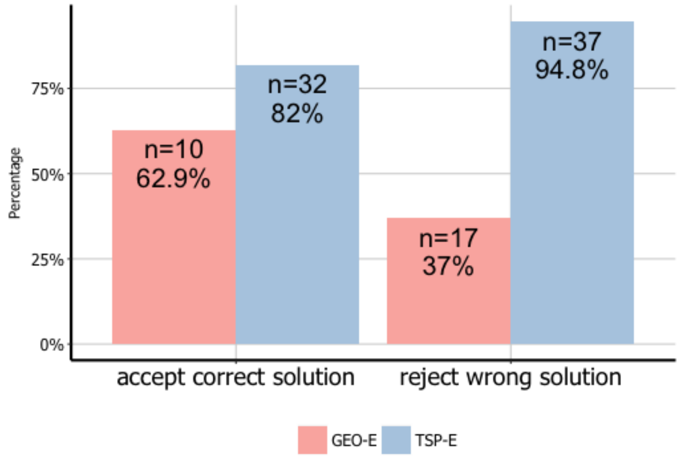

Figure B: Non-Solvers’ acceptance and rejection of solutions for TSP-E and GEO-E

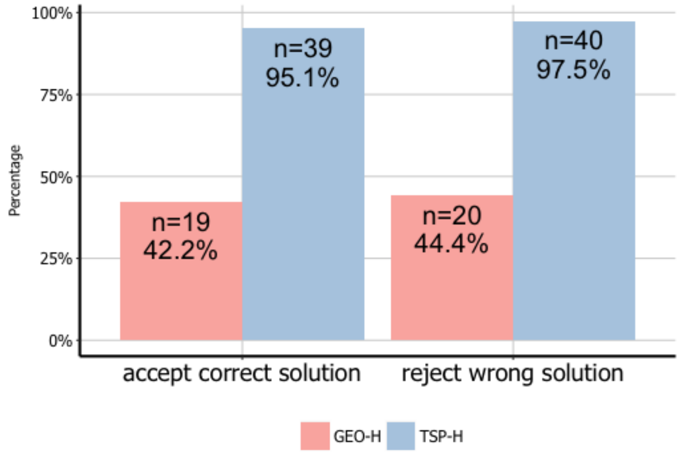

Figure C: Non-Solvers’ acceptance and rejection of solutions for TSP-H and GEO-H in BGU

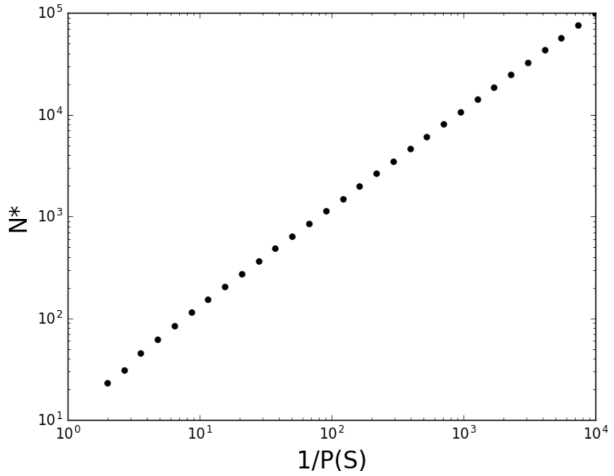

Figure D: The optimal group size (N^*) scales inversely to the difficulty of the problem ($P(S)$). The relationship between N^* and $1/P(S)$ is shown on a log-log scale.
