# Supplementary material for: The more the merrier? Increasing group size may be detrimental to decision-making performance in nominal groups
Source: PLoS One. 2018 Feb 27;13(2):e0192213. doi: 10.1371/journal.pone.0192213 (PMC5828441; doi:10.1371/journal.pone.0192213)
Supplement: S2 File — Consent form informed consent to participate in the online study. (PDF) [file pone.0192213.s002.pdf]

3. Consent Form

Please consider this information carefully before deciding whether to accept this task.

PURPOSE OF RESEARCH: To examine problem solving performance.

WHAT YOU WILL DO: You will be asked to solve a graph puzzle.

TIME REQUIRED: Participation will take approximately 10 minutes.

RISKS: There are no anticipated risks associated with participating in this study. The effects of participating should be comparable to those you would experience from viewing a computer monitor for 20 minutes and using a mouse.

COMPENSATION: Upon completion of this task, you will receive a code to enter on the Amazon Mechanical Turk task page, and you will receive the amount that was indicated on the task page.

CONFIDENTIALITY: Your participation in this study will remain confidential. Your responses will be assigned a code number. You will NOT be asked to provide your name. You will be asked to provide your age and gender. Throughout the experiment, we may collect data such as browser type, operating system version, mouse movements, and error rates.

PARTICIPATION AND WITHDRAWAL: Your participation in this study is voluntarily, and you may withdraw and return the task to Amazon Mechanical Turk at any time. You will receive a compensation only if you complete the task. You may withdraw at any time by closing the web page of the task.

AGREEMENT: The nature and purpose of this research have been sufficiently explained and I agree to participate in this study. I understand that I am free to withdraw at any time.

☐ I agree and will participate in this study.
