# Supplementary material for: The more the merrier? Increasing group size may be detrimental to decision-making performance in nominal groups
Source: PLoS One. 2018 Feb 27;13(2):e0192213. doi: 10.1371/journal.pone.0192213 (PMC5828441; doi:10.1371/journal.pone.0192213)
Supplement: S3 File — Instructions for subjects in the GEO condition. (PDF) [file pone.0192213.s003.pdf]

# 4. Instructions to subjects: GEO condition

The "Dead End Road" is a two-player game, played by a blue player and a green player.

The game's opening screen is shown below.

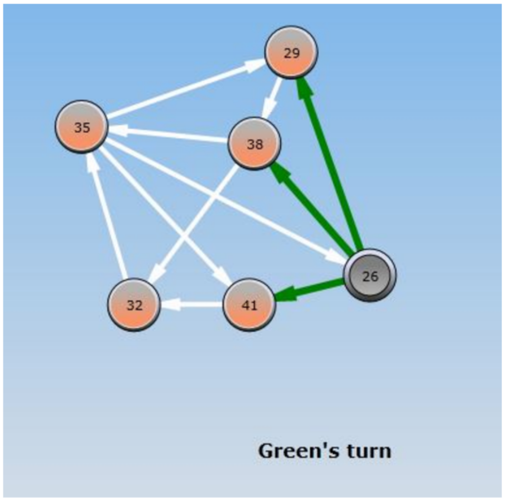


Players alternate turns, where at each turn the active player chooses a vertex that is connected to the vertex chosen in the previous turn.

For example, the image below, vertex 26 is the initial vertex.

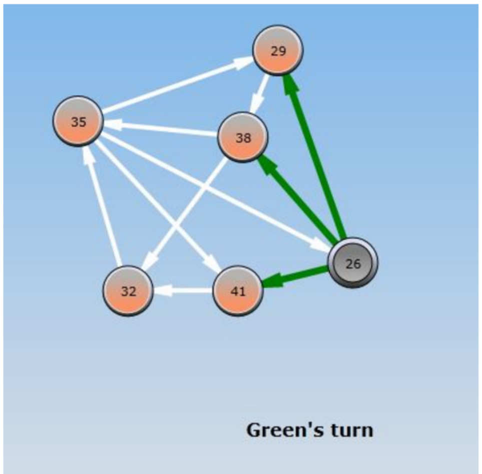


You will be the first player to choose a vertex.

The starting vertex is colored in grey. You should choose one of the vertices that can be reached by one of the green arrows.

### The Goal

A player wins the game by choosing such that the other player is "stuck" at a dead end, that is, there are no vertices that can be chosen.

**An example of a situation where the player Green won:** You can see the last vertex, vertex 32, has arrows going out but since all the destinations of vertex 32 points have been selected, this is a dead end vertex.

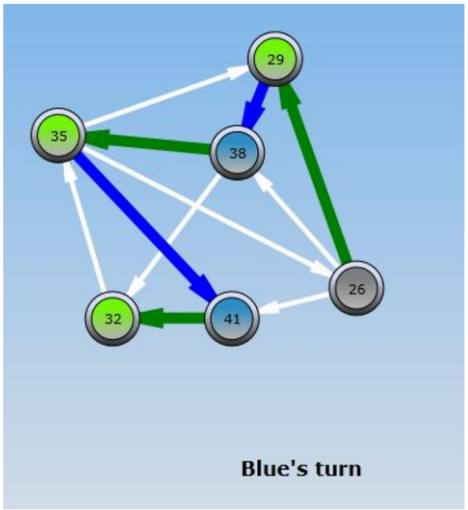


When there are green arrows, it is your turn to choose a vertex. When blue arrows appear, it is your opponent's turn.

In your turn, choose a vertex that can be reached by a green arrow, by clicking the vertex

The player whose turn to play is displayed at the bottom.

The image shows a phase of the game when it is the opponent's turn to choose a vertex (the blue player)

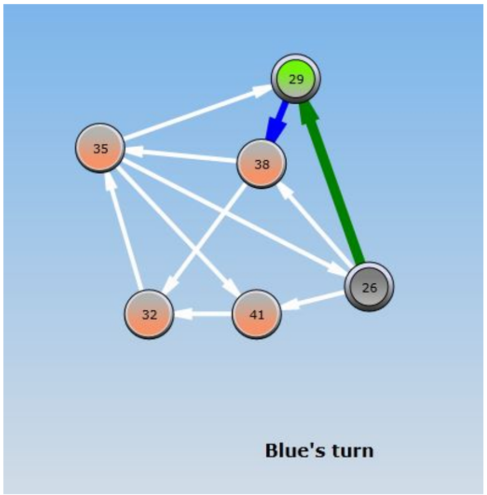


### The Task

In this game you will be the first player (green). Your goal is to choose vertex that guarantees a victory, regardless of the opponent's choices.

You can simulate your steps and the steps of the opponent on the graph using the interface described earlier.

**It is important to highlight that in the interface you play both players. Namely, you select vertices for the green player, and also blue player.**

When there are green arrows, it is your turn to choose a vertex. When blue arrows appear, it is your opponent's turn.

### Submit your answer

At any time, you can select at the bottom of the page the first vertex which will guarantee that you (the green player) wins, as follows:

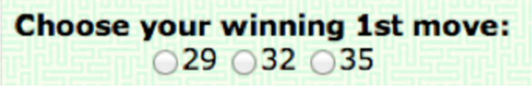


At any stage, you can backtrack as many steps as you want by clicking the "UNDO MOVE" button, or by clicking on the vertex you selected last.

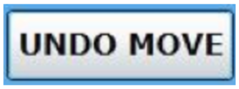


You can also click the "RESET" to get a clean graph to clear the graph from all the moves that were chosen.

### Voting on solutions offer by others

After you submit your solution, you will be presented with solutions to this game that were proposed by other people. You will be asked to determine, for each solution, whether it is correct or incorrect.

The solution will be presented by displaying the first vertex that was chosen by the participant. You can use the interface in order to examine the proposed solution and decide whether it is correct.

The interface is not automatically reset and it will display the graph at the last state from your solution. You can reset the graph by clicking the "reset" button or undoing your previous moves as described earlier.

### Your payment:

- 70 cents fixed payment
- 35 cents for solving the problem correctly.
- 35 cents for determining the correctness of each of the solutions presented.

**When you are ready to start the task, press "continue". You will then complete a short comprehension test and will be presented with the problem.**
